# Early-onset preeclampsia is characterised by an increased vascular tone in internal jugular veins

**DOI:** 10.3389/fcvm.2022.911059

**Published:** 2022-08-12

**Authors:** Inge Dierickx, Cécile Kremer, Liesbeth Bruckers, Wilfried Gyselaers

**Affiliations:** ^1^Department of Obstetrics and Gynaecology, Sint Lucas Ziekenhuis, Gent, Belgium; ^2^Department of Physiology, Hasselt University, Hasselt, Belgium; ^3^Interuniversity Institute for Biostatistics and Statistical Bioinformatics, Hasselt University, Hasselt, Belgium; ^4^Department of Obstetrics and Gynaecology, East Limburg Hospital, Genk, Belgium

**Keywords:** combined Doppler-Electrocardiogram, venous pulse transit time, venous impedance index, preeclampsia, gestational hypertension, venous maternal haemodynamics, eclampsia, internal jugular vein

## Abstract

**Purpose:**

This study aimed to investigate Doppler characteristics of maternal internal jugular veins in uncomplicated pregnancies vs. those affected by hypertensive disorders.

**Materials and methods:**

Venous pulse transit time and venous impedance index were measured at three different locations (right proximal, right distal, left proximal) of internal jugular veins according to a standardised combined Doppler-Electrocardiogram protocol in five different groups of pregnant women: uncomplicated pregnancy, early-onset preeclampsia, late-onset preeclampsia, gestational hypertension, and normotensive pregnancies with a small for gestational age foetus. Values of both parameters of the latter four groups were plotted against the reference range of uncomplicated pregnancies at corresponding gestation. Linear mixed models with random intercept were used to investigate gestational changes in venous pulse transit time and venous impedance index at the three internal jugular vein locations within and between the different groups.

**Results:**

A total of 127 women were included: 41 had uncomplicated pregnancies, 13 had early-onset preeclampsia, 25 had late-onset preeclampsia, 22 had gestational hypertension, and 26 had normotensive pregnancies with a small for gestational age foetus. Venous pulse transit time values were lower than uncomplicated pregnancy (*p* ≤ 0.001) at all three locations in the third trimester of early-onset preeclampsia.

**Conclusion:**

Contrary to late-onset preeclampsia and gestational hypertension, early-onset preeclampsia is characterised by a lower venous pulse transit time at internal jugular veins compared to uncomplicated pregnancy, suggesting increased venous vascular tone.

## Introduction

Gestational hypertensive disorders occur in 5–8% of all pregnancies, accounting for both possibly serious maternal and foetal/neonatal morbidity and mortality (1). This obstetric disorder is characterised by cardiovascular maladaptation, not only at the heart and the arterial vascular tree but also in the venous compartment, as was reported for the first time by Bateman et al. (2).

A combined Doppler-Electrocardiogram (CDE) assessment provides a safe, cheap, easily accessible, and non-invasive tool to explore maternal venous haemodynamics (3, 4). Venous pulse waves indirectly reflect right cardiac atrial function and can be detected at supra- and infracardiac levels (5). The atrial contraction (ECG P-top) is responsible for a retrograde rebounce of blood into the venous tree (6) which is reflected by the A-deflection in the pulse waveform (7). The electro-mechanic delay between the ECG P-top and the corresponding Doppler A-deflection can be defined as the P–A interval. This heart rate-dependent interval can be corrected by using the P–A interval/R–R interval ratio also referred to as the venous pulse transit time (VPT). VPT is considered the venous equivalent of the arterial pulse transit time and is inversely related to vascular wall stiffness. In conditions of increased vascular tone or wall stiffness, the propulsion wave is transported faster through the circulation than in conditions of low vascular tone, being responsible for a decrease in VPT (8–10).

The venous impedance index (VI), calculated by X-A/X, is the Doppler equivalent of the arterial pulsatility index (11), representing the intracycle variation of blood flow velocities (12) and can be considered a proxy for distensibility and/or compliance. A large value indicates a strong intravenous rebound of atrial contraction, which counteracts venous drainage from the organs and a decrease of VI is consistent with an increase in compliance (13).

Previous studies have shown that VPT increases and VI decreases (flattening of the pulse waveform) with gestational age at interlobar renal, hepatic and internal jugular veins (IJVs) (3, 14). Uncomplicated pregnancy (UP) is thus characterised by a gradual reduction in vascular tone and an increase in vascular compliance when pregnancy advances. On top of that, Gyselaers et al. have described that short VPT and high VI at hepatic and renal interlobar veins are characteristic features of preeclampsia (PE) (15, 16), presenting more pronounced in early than in late PE (17), but are not observed in GH (18).

PE can include cardiovascular changes, hematologic abnormalities, hepatic and renal impairment, and neurologic manifestations (19). However, >50% of (pre) eclampsia-related maternal deaths are attributed to cerebrovascular events and are thereby by far the most important direct cause of death (20).

Within the complex system of cerebral haemodynamics, the venous heart-brain axis has become the focus of non-obstetric studies in the last few years, not only in confirming its role in cerebrovascular haemodynamics but also in investigating its hypothesised involvement in the pathophysiology of neurovascular and -degenerative disorders (21, 22).

The goal of this study was to evaluate whether VPT and VI at IJVs are different in pregnant women with hypertensive disease relative to the findings in UPs.

## Materials and methods

### Ethics statement

Ethical approval by the Local Ethics Committee was given before study onset (study reference 2011–12 and 2016–41) and written informed consent was obtained from all participants. All procedures were in accordance with institutional guidelines and adherent to the principles of the Declaration of Helsinki.

### Participants

A total of five groups of women with singleton pregnancies were included: (1) women with UP and birth of a normal or large gestational age baby according to gender and parity specific customised local reference charts. For inclusion, women with singleton pregnancies presenting in the first trimester at the outpatient antenatal clinic of the Sint Lucas Hospital in Ghent were invited to participate in this study. These women were evaluated prospectively at eight consequent moments during their pregnancy (12-16-20-24-28-32-36 and 38 weeks). (2) For “complicated” pregnancies, women were invited to participate in diagnosis at the outpatient antenatal clinic of the Sint Lucas Hospital in Ghent. These women with a “complicated” pregnancy were categorised into 4 groups: early-onset preeclampsia (EPE), late-onset preeclampsia (LPE), GH, and normotensive pregnancies with small for gestational age baby (SGA). For SGA, women with singleton pregnancies presenting in the outpatient antenatal clinic of the Sint Lucas Hospital in Ghent were invited to participate when the sonographically estimated foetal weight during the course of pregnancy was below the 10th percentile on the foetal growth chart, and when birth weight < P10 was confirmed after birth. Whenever possible, consecutive measurements with an interval ≥ 1 day were performed on the same woman in this group of “complicated” pregnancies. Both for the UP group and the group of “complicated” pregnancies, women with pre-existing maternal or gestational diseases (e.g., with pre-existing cardiac, renal, liver, hematologic auto-immune diseases or a history of migraine or thyroid, neck, or brain surgery) were excluded.

At birth, data on gestation outcomes were categorised according to the criteria by the International Society for Studies of Hypertension in Pregnancy. PE was defined as new-onset hypertension with proteinuria ≥ 300 mg/24 h, other organ dysfunction, or foetal growth restriction, and is labelled as early-onset at clinical presentation < 34 weeks (EPE) and late-onset at presentation ≥ 34 weeks (LPE). Next to proteinuria ≥ 300 mg/24 h, a urine protein-to-creatinine ratio (mg protein/mg creatinine) higher than 0.3 was also considered to be diagnostic for significant proteinuria. GH was defined as new-onset hypertension without proteinuria, other organ dysfunction, or foetal growth restriction. Women who gave birth to a neonate with birth weight <10th percentile according to gender and parity-specific customised local reference charts after a normotensive pregnancy were classified as SGA (23). Line Customised birth weight charts were established from a cohort of 34,684 neonates, born as singletons without congenital anomalies in Sint Lucas Hospital Ghent between 2000 and 2013. Charts were categorised into four groups: primiparous baby girl, primiparous baby boy, multiparous baby girl, and multiparous baby boy. Birth weights were classified per week of gestation, and birth weight percentiles were calculated with an interval of 2.5% between P2.5 and P97.5. According to these population-specific data, the weight at birth of each neonate in the study was expressed as a customised birth weight percentile.

Next to gestation outcome, the following maternal data were collected: age, parity, smoking, pregestational body mass index (BMI) as per medical history, weight gain, and medication (non-cardiovascular, antihypertensive).

### Combined Doppler-Electrocardiogram assessment of internal jugular veins

In agreement with the previous study, CDEs of IJVs were performed at three different locations (right proximal, right distal and left proximal according to the previously described standardised protocol with acceptable reproducibility and repeatability (equipment: Toshiba Nemio XG; 6- to 11-MHz linear-array transducer) (24). To summarise, the participant was examined in the supine position and was asked to hold their head stable in a neutral position by facing a focus point at the ceiling during the entire examination. The impact of Valsalva manoeuvres and breathing, thorax wall, and diaphragm movements on blood flow was demonstrated and the importance of breath holding was explained. As soon as the triphasic waveform of the IJV was detected by pulsed Doppler imaging, the participant was instructed to hold her breath at the end of a normal expiration.

In all women, four consecutive measurements were performed at the three IJV locations by the principal investigator (ID). Previous research has shown that averaging four replicates stabilised the CDE IJV measurements and bootstrap analysis suggested that increasing the number of replicates beyond four will not elicit further improvements in the stability of the averaged values (24).

The ultrasound images, with encrypted participant details, were transferred from a portable device to the hard disk of a computer for offline assessment. Irfan View (Version 64 4.41, @ 1996–2016 by Irfan Skiljan, Jajce, Central Bosnia Canton, Bosnia) was used to mark and measure the flow velocities at the Doppler A- and X-deflection, the P-A interval, and R-R interval. VPT and VI were calculated as P–A interval/R–R interval ratio and (X-A)/X for each location at each session.

### Statistical analysis

Demographic data are presented as median (IQR) or N (%), and the difference between the five groups was assessed using Kruskal-Wallis's test for continuous variables and Fisher's exact test for categorical variables.

Linear mixed models with random intercepts to account for variation between women and to correct for dependence between different measurements from the same woman were used to investigate gestational changes of VPT and VI at the three IJV locations within and between the different groups. Comparisons between groups (EPE vs. UP, LPE vs. UP, GH vs. UP, and SGA vs. UP) were performed at corresponding UP gestational age. For both outcomes, it was investigated whether there was a linear, quadratic, or cubic evolution during the pregnancy and whether this evolution was different for the two groups. Model selection was done using backward elimination, i.e., non-significant terms were removed from the model in a stepwise manner. All analyses were performed using SAS^®^ version 9.4 (SAS Institute, Cary NC).

## Results

### Participants

A total of 54 women were assessed for the group UP of which 13 women were excluded. Among the participants, two women developed gestational diabetes and seven women were diagnosed with hypertensive disease during the course of their pregnancy (2 EPE, 2 LPE, and 3 GH). Four women unexpectedly gave birth to an SGA neonate, leaving 41 participants eligible for UP inclusion. For UP, a total of 22/328 (41 participants x 8 moments in pregnancy) assessments were not performed. 13/22 assessments (6 at 12 weeks, 2 at 16, 20, and 24 weeks, and 1 at 36 weeks) were not performed due to interruption of the procedure due to a medical emergency in another patient or practical patient or investigator organisation. 9/22 women delivered between 36 and 38 weeks, leaving 306/328 eligible for inclusion. A total of 86 women with a “complicated” pregnancy were included. Whenever possible, consecutive measurements within the same woman were performed in this latter group: EPE: 29 measurements in 13 women; LPE: 44 measurements in 25 women; GH: 38 measurements in 22 women; SGA: 84 measurements in 26 women. Antihypertensive treatment had already been initiated before the CDE IJV assessment in 19/29 (66%), 18/44 (41%), and 18/38 (47%) participants for EPE, LPE, and GH respectively.

Maternal data and data on gestation outcomes are presented in Table 1. Pregestational BMI was higher in GH, and there were more smokers in SGA than in UP. Women in EPE, LPE, and GH delivered earlier, and the birth weight was significantly lower in EPE, LPE, and SGA than in UP. In LPE, fewer male infants were born compared to UP.

**Table 1:** Maternal data and data of gestation outcome among women with UP, EPE, LPE, GH, and SGA.

	**UP**	**EPE**	**LPE**	**GH**	**SGA**	***p*-value**
*n* (women)	41	13	25	22	26	
*n* (assessments)	306	29	44	38	84	
Maternal age, years	30(28–32)	31(27–32)	28(27–31)	31(28–34)	30(26–32)	.488
Nulliparity, *n* (% women)	22(54)	11(85)	19(76)	16(73)	15(57)	0.146
Cigarette smoker, *N* (% women)	0(0)	0(0)	2(8)	0(0)	8(31)^*^	<0.001
Pregestational BMI, kg/m^2^	22(20.9–24.2)	22.8(20.8–26)	24.8(21.3–28.4)	25.7(23.2–28.8)^*^	21.8(19–24.3)	0.003
Maternal weight gain, kg	11(10–14)	12(9–18)	13(11–16)	13(10–16)	10(9–13)	0.093
Non-cardiovas-cular medication, *N* (% women)	3(7)	1(8)	0(0)	3(14)	1(4)	.353
Antihypertensive medication, *N* (% women)	0(0)	8(62)^*^	4(16)	9(41)^*^	0(0)	< .001
Male gender (%)	19(46)	4(30)	3(12)^*^	11(50)	10(38)	.029
gestational age at delivery, weeks	39.4 (39–40.3)	34 (33.1–35.3)^*^	37.5 (36,2–39.4)^*^	38.3 (37.1–39.5)^*^	38.5 (38–39.8)	< .001
birth weight, G	3,360 (3,100–3,545)	2,005 (1,510–2,400)^*^	2,600 (2,120–3,465)^*^	3,080 (2,549–3,373)	2,595 (2,429–2,858)^*^	<0.001
birth weight, percentile	35 (25–70)	18 (5–55)	13 (3–60)	38 (11–53)	5 (3–8)^*^	<0.001

Data are presented as n (%) or median (interquartile range). P-value gives the overall significance whereas an ^*^ indicates a significant difference compared to UP.

### Statistical analysis

**Figure 1:**
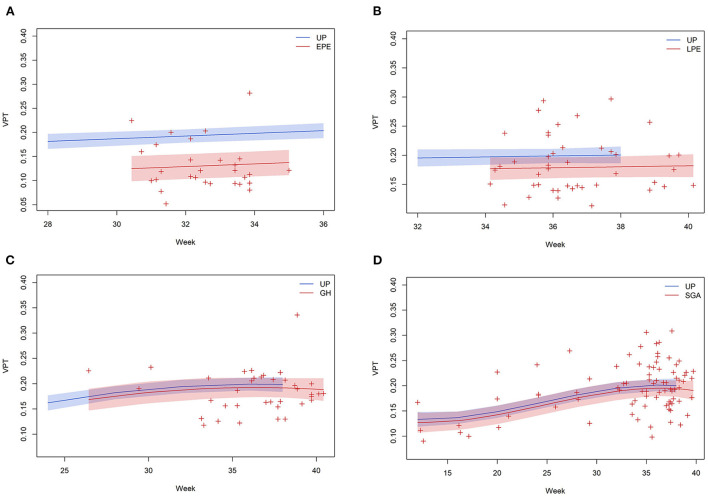
Evolution of VPT as measured at right distal IJV in EPE **(A)**, LPE **(B)**, GH **(C)**, and SGA **(D)** relative to gestational age and plotted against the reference values of 41 UPs.

For VPT, an increasing trend with conversion to a decreasing trend at approximately 35 weeks was observed during UP at all three IJV locations. Only in EPE, were VPT values significantly lower than UP at all IJV locations (*p* ≤ 0.001). EPE showed a linear increasing trend at right proximal and right distal IJV from 31 to 34 weeks of gestation (Figure 1A), no significant trend was found at left proximal IJV. With exception of GH at the right proximal IJV, the VPT trends of LPE, GH, and SGA were similar to UP at the corresponding gestational age (Figures 1B–D). In GH at the right proximal IJV, VPT showed a decreasing trend with conversion to an increasing trend at around 33–34 weeks of gestation.

For VI, during UP there was an increasing trend with conversion to a decreasing trend at ~22–23 weeks at all three IJV locations. No difference was found between the gestational evolution of VI in EPE, LPE, GH, and SGA compared to UP, with exception of GH at right proximal IJV and SGA at right distal IJV. In GH at the right proximal IJV, an increasing trend with conversion to a decreasing trend at a gestational age of around 33–34 weeks was observed. In SGA at right distal IJV, a decreasing trend with conversion to an increasing trend was observed at a gestational age of around 33 weeks. Absolute values of VI were not different between EPE vs. UP, LPE vs UP, GH vs. UP, and SGA vs. UP, with exception of SGA at left proximal IJV where they were significantly lower (*p* = 0.009).

## Discussion

In this paper, characteristics of the jugular venous pulse waveform obtained by CDE in pregnant women with hypertensive disorders are described. To the best of our knowledge, this study is the first to compare Doppler characteristics at IJVs in pregnant women with different clinical types of hypertensive disease relative to the findings in UPs.

### Main findings

For CDE IJV assessment during pregnancy complicated by different clinical types of hypertensive disease, the key findings are:

(1) Only in EPE, absolute VPT values are significantly lower than in UP.
(2) In pregnancies complicated with different clinical types of hypertensive disease, VPT at supracardiac level shows similar patterns to in previous studies on VPT at the infracardiac level.
(3) VI assessments at the supracardiac level seem to be less informative than those at the infracardiac level.

### Strengths and limitations

The strengths of this study are the use of a rigid standard protocol for CDE IJV assessment by only one investigator with known intra- and inter-observer correlation (24) and the use of customised population-specific birth weight charts. Thanks to the complete and directly available demographic data, the included women in each subgroup strictly complied with all reported criteria of the International Society for Studies of Hypertension in Pregnancy. Additionally, they were free of complicating maternal or gestational diseases at the time of inclusion.

This study has some limitations. Firstly, we acknowledge the relatively low number of included patients per group. Consequently, the statistical power of this study is <80% and the present findings need to be confirmed by larger prospective studies. Secondly, this technique demands a highly trained investigator and is subject to both patient- and investigator-dependent artefacts, as described previously (3, 24). Thirdly, previous research has shown that cardiovascular adaptations appear to be enhanced by a subsequent pregnancy and that parous women without a history of preeclampsia have a lower peripheral vascular resistance than nulliparous women (25, 26). In this study, the difference in the proportion of nulliparity between UP en EPE is only marginally not significant (*p* = 0.057) and may have been a confounding factor, especially for VPT values. Larger prospective studies are recommended. Fourthly, this study does not allow drawing any conclusion on the possible impact of starting antihypertensive medication on the characteristics of the jugular pulse waveform. Ideally, the use of antihypertensive medication should be excluded, however, this would reduce the number of inclusions even further as antihypertensive treatment is the standard of care from the moment the blood pressure becomes elevated. Fifthly, the included women were not routinely screened for thrombophilia or auto-immune diseases. Exclusion of this study was only made on the women's medical history. Similarly, in this small observational study, interference from a variety of variables such as smoking, coffee use, and other health behavioural or cultural influences cannot be entirely excluded. For this, a large prospective study is needed. Finally, this study does not allow drawing conclusions about possible interfering variables within the complex functioning of cerebral outflow as reflected in the maternal jugular venous waveform.

### Interpretation

In this study, VPT at IJVs in EPE is significantly lower than in UP. No difference in absolute values was found in LPE, GH, and SGA relative to UP. These observations are in line with previous studies at the infracardiac level. At the level of the kidneys, VPT is short already weeks before the clinical onset of EPE (27). In LPE, borderline reduced VPT has been reported, usually unilaterally and presenting only at the onset of disease (27). At the liver, a significantly short VPT in the clinical stage of EPE is observed (17). The same is true for LPE, but to a much lesser degree (27). GH presents without abnormalities of VPT at renal and hepatic levels (16).

Despite the detailed scanning protocol, VI assessments at IJVs show high variability and absence of significant trends at most of the three IJV locations. This is in contrast with previous studies where VI in liver and kidney were higher in PE compared to UP. Gyselaers et al. reported a higher VI in EPE than in LPE at the infracardiac level, and this higher value of VI was present weeks before the clinical symptoms (17), in contrast with LPE (27). Former studies of this research team at the infradiafragmatic level mainly focused on intraparenchymatous vessels in the liver and kidneys. This prevents external venous compression by the ultrasound probe. Due to anatomic reasons, this problem cannot be tackled at the jugular veins and is responsible for higher intra-and inter-observer variations above than below the diaphragm, larger standard deviations, and measurement ranges as well as more failed samplings (3, 24). This study adds to the reported dysfunction of the venous system in PE, but not in GH, at the supracardiac level. This is in agreement with previous studies at the infracardiac level.

This study does not allow us to draw any conclusions on the possible role of the venous heart-brain axis in the pathophysiology of cerebral complications due to gestational hypertensive disease. Cerebral circulation has an autoregulatory mechanism to maintain a steady cerebral blood flow. Severe hypertension can overwhelm this system and result in blood-brain barrier dysfunction (28) however the degree of hypertension may not always predict the risk of eclampsia (29). In addition to hypertension, recent studies suggest that the cerebrovenous system and cerebral outflow may be important factors in guaranteeing normal brain function. The cerebrovenous system is a complex three-dimensional structure that is often asymmetric and represents a much more variable pattern than the arterial anatomy. The connexion between the right atrium and vena cava is valveless, but dysfunction of IJV valves can cause an increase in the mean value for cerebral venous pressure (30, 31). Intracranial venous hypertension, according to Talbert, may trigger a chain of events involving endothelial dysfunction, increased permeability of the blood-brain barrier, and extravasation of colloids (32). Our results of enhanced venous pulse transit in early-onset preeclampsia are in line with a generalised state of endothelium activation at both the arterial and venous sites.

## Conclusion

This study adds to the reported dysfunction of the venous system in PE at both supra- and infracardiac levels. Again, this association is lacking in GH. Short IJV pulse transit time in EPE is consistent with an increased venous vascular tone. This observation opens perspectives to target cerebral venous haemodynamics in the prevention and treatment of preeclampsia-related brain dysfunction.

## Data availability statement

The raw data supporting the conclusions of this article will be made available by the authors, without undue reservation.

## Ethics statement

The studies involving human participants were reviewed and approved by Commissie voor medische ethiek, AZ Sint Lucas Gent, Belgium. The patients/participants provided their written informed consent to participate in this study.

## Author contributions

ID: data collection, interpretation results, and wrote the manuscript. CK and LB: statistical analysis of data, preparation figures, and helped to draft the manuscript. WG: design and coordination of the study, interpretation results, and helped to draft the manuscript. All authors contributed to the article and approved the submitted version.

## Conflict of interest

The authors declare that the research was conducted in the absence of any commercial or financial relationships that could be construed as a potential conflict of interest.

## Publisher's note

All claims expressed in this article are solely those of the authors and do not necessarily represent those of their affiliated organizations, or those of the publisher, the editors and the reviewers. Any product that may be evaluated in this article, or claim that may be made by its manufacturer, is not guaranteed or endorsed by the publisher.

## Abbreviations

BMI: body mass index
CDE: Combined Doppler-Electrocardiogram
EPE: early-onset preeclampsia
GH: gestational hypertension
IJV: internal jugular vein
LPE: late-onset preeclampsia
PE: preeclampsia
SGA: normotensive pregnancy with small for gestational age baby
VI: venous impedance index
VPT: venous pulse transit time.
